# Prevalence of hepatitis B and hepatitis C viral infections in various subtypes of B-cell non-Hodgkin lymphoma: confirmation of the association with splenic marginal zone lymphoma

**DOI:** 10.1038/bcj.2017.28

**Published:** 2017-03-31

**Authors:** W Xiong, R Lv, H Li, Z Li, H Wang, W Liu, D Zou, L Qiu, S Yi

**Affiliations:** 1 State Key Laboratory of Experimental Hematology, Department of Lymphoma and Myeloma, Institute of Hematology and Blood Disease Hospital, Chinese Academy of Medical Sciences and Peking Union Medical College, Tianjin, China

Non-Hodgkin lymphoma (NHL) ranked as the sixth most common cancer in 2016.^1^ Its incidence has steadily increased in the past decade. Both environmental and genetic factors can promote the development of NHL. It has been hypothesized that chronic antigenic stimulation, especially due to a virus such as the human immunodeficiency virus, Epstein-Barr virus and hepatitis C virus (HCV), plays an important role in the pathogenesis of NHL. Previous data indicated that hepatitis B virus (HBV) infection is associated with B-cell NHL (B-NHL) in HBV-prevalent areas such as Japan and Korea.^2, 3^ However, studies that systematically evaluate the prevalence of HBV and HCV infections in each B-NHL subtype are rare. In this study, we aimed to illustrate the distribution of hepatitis virus infection in a large series of B-NHL patients in China, a region in which hepatitis virus infection is prevalent.

In total, 733 newly diagnosed indolent patients and 148 aggressive B-NHL patients with integrated HBV and HCV results were unselectively enrolled in this study. The diagnosis criterion was in accordance with the World Health Organization Classification of Tumors of Hematopoietic and Lymphoid Tissues.^4^ The B-NHL subtypes are shown in Table 1. HBs-Ag and anti-HCVAb-positive patients were considered to have HBV and HCV infection, respectively. To compare the hepatitis viral infection data with that of the normal population, we referenced the recent epidemiological investigation of the hepatitis viral infection in China, which enrolled 81 775 residents with HBV and 78 746 residents with HCV.^5, 6^

With regard to HBV infection status, 9.0% of the patients (79/881) were HBs-Ag-positive, which was significantly higher than the proportion in the general population,^5^ as shown in Table 1. Specifically, a significantly higher prevalence of HBV infection in patients with aggressive B-NHL was observed compared to both the indolent B-NHL group and the general population. The subtype analysis of the aggressive group is shown in Table 1. The presence of HBs-Ag was comparable in the indolent B-NHL and the general population. Unexpectedly, among all the indolent B-NHL subtypes, 9 of the 48 (18.8%) splenic marginal zone lymphoma (SMZL) patients were HBs-Ag-positive. A positive association was observed between SMZL and HBs-Ag seropositivity. Furthermore, compared to other indolent B-NHL subtypes, the SMZL group also had a significantly higher HBs-Ag infection rate (18.8 vs 7.1%, *P*=0.005).

The prevalence of HCV infection in the subtypes of B-NHL is also shown in Table 1. Compared to the general population,^6^ the anti-HCV-Ab-positive rate was significantly higher in the entire B-NHL group and the indolent B-NHL group. Only two mantle cell lymphoma patients had an HCV infection in the aggressive B-NHL. However, HCV infection was universal in the indolent B-NHL group. Remarkably, the SMZL group had the highest HCV infection rate.

This is the first study to evaluate the HBV and HCV infection rate in each subtype of B-NHL systematically in a large series. We found that not only HCV but also HBV seropositivity was associated with some but not all B-NHLs. Due to the geographical and epidemiological variability as well as different lymphoma morbidity, the results varied across areas. However, an increasing number of reports support a positive association of HCV with some subtypes of B-NHL, such as DLBCL and SMZL,^7, 8^ which is similar to our findings. Among all the studies, the most convincing results are that SMZL patients with HCV infection who received only anti-HCV therapy would achieve complete or partial remission.^8, 9^ Nevertheless, whether HBV would also have an association with B-NHL subtypes, especially SMZL, has rarely been studied. Our study reported a significantly positive association between HBV and SMZL, with an approximately threefold risk compared to the general population. This association has not been reported previously. Most of the previous studies were only case reports that showed that SMZL patients would achieve remission with HBV-eradicating therapies.^10, 11^ Most recently, Romanian researchers^12^ reported a high prevalence of HBV in SMZL patients, 17.7% of whom were HBs-Ag-positive, which is similar to our findings. Although only 17 patients had complete hepatitis viral infection data in this study, the high seroprevalence of HBs-Ag among SMZL patients implies a certain association between this virus and SMZL in HBV-prevalent areas.

In conclusion, we confirm that hepatitis virus infection differs in B-NHL subtypes. The prevalence rate of both HBV and HCV in SMZL patients was higher than that of the general population and other indolent B-NHL subtypes, suggesting that hepatitis virus infection might play an etiologic role in SMZL pathogenesis.

## Figures and Tables

**Table 1:** Prevalence of HBV and HCV infection in patients with different types of B-cell non-Hodgkin lymphoma

*Category*	*Total* (N)	*HBs-Ag infection rate*		*Unadjusted odds ratio (95% CI)*	P *value*	*HCV infection rate*		*Unadjusted odds ratio (95% CI)*	P *value*
		N	*%*			N	*%*		
General population	81 775	5888	7.2	Reference					
General population	78 746					316	0.40	Reference	
B-NHL patients	881	79	9.0	1.270 (1.006-1.602)	**0.044**	16	1.8	4.606 (2.775-7.645)	**0.000**
Indolent B-NHL	733	58	7.9	1.107 (0.846-1.450)	0.458	14	1.9	4.848 (2.824-8.322)	**0.000**
Aggressive B-NHL	148	21	14.2	2.131 (1.342-3.384)	**0.001**	2	1.35	3.411 (0.841-13.828)	0.068

*According to indolent lymphoma subtype*									
SMZL	48	9	18.8	2.974 (1.440-6.143)	**0.002**	2	4.2	10.826 (2.617-44.788)	**0.000**
CLL	279	21	7.5	1.049 (0.672-1.638)	0.833	6	2.2	5.472 (2.419-12.380)	**0.000**
LPL/WM	119	6	5.1	0.684 (0.301-1.556)	0.363	3	2.6	6.439 (2.036-20.366)	**0.000**
FL	74	6	8.1	1.137 (0.493-2.621)	0.763	0	0		0.586
B-LPDu	132	10	7.5	1.056 (0.554-2.014)	0.868	1	0.76	1.901 (0.265-13.636)	0.516
HCL	33	4	12.5	1.778 (0.625-5.058)	0.274	1	3.0	7.781 (1.060-57.116)	**0.017**
NMZL	27	3	11.1	1.611 (0.485-5.352)	0.432	1	3.7	9.576 (1.296-70.786)	**0.007**
MALT	15	0	0		0.281	0	0		0.806
B-PLL	6	0	0		0.495	0	0		0.877

*According to aggressive lymphoma subtype*									
DLBCL	58	12	20.7	3.362 (1.780-6.350)	**0.000**	0	0		0.629
MCL	59	8	13.6	2.022 (0.959-4.262)	0.059	2	3.5	9.054 (2.199-37.279)	**0.000**
Burkitt lymphoma	2	0	0		0.694	0	0		0.929
B-LBL	14	0	0		0.297	0	0		0.813
Aggressive B-NHL unclassified	15	1	6.7	0.921 (0.121-7.002)	0.936	0	0		0.806

Abbreviations: B-LBL, B-cell lymphoblastic lymphoma; B-LPDu, unclassified B-cell chronic lymphoproliferative disorders; B-NHL, B-cell non-Hodgkin Lymphoma; B-PLL, B-cell prolymphocytic leukemia; CLL, chronic lymphocytic leukemia; DLBCL, diffuse large B-cell lymphoma; HBV, hepatitis B virus; HCL, hairy cell leukemia; HCV, hepatitis C virus; FL, follicular lymphoma; LPL/WM, lymphoplasmacytic lymphoma/Waldenström macroglobulinemia; MALT, mucosa-associated lymphoma; MCL, mantle cell lymphoma; NMZL, nodal marginal zone lymphoma; SMZL, splenic marginal zone lymphoma. *P* value comes from comparison with general population; bold indicates significant difference (*P*<0.05).
